# Interactive Tele-Radiological Segmentation Systems for Treatment and Diagnosis

**DOI:** 10.1155/2012/713739

**Published:** 2012-05-07

**Authors:** S. Zimeras, L. G. Gortzis

**Affiliations:** ^1^Department of Statistics and Actuarial-Financial Mathematics, University of the Aegean, Samos, 83200 Karlovassi, Greece; ^2^School of Medicine, University of Patras, Patras, Greece

## Abstract

Telehealth is the exchange of health information and the provision of health care services through electronic information and communications technology, where participants are separated by geographic, time, social and cultural barriers. The shift of telemedicine from desktop platforms to wireless and mobile technologies is likely to have a significant impact on healthcare in the future. It is therefore crucial to develop a general information exchange e-medical system to enables its users to perform online and offline medical consultations through diagnosis. During the medical diagnosis, image analysis techniques combined with doctor's opinions could be useful for final medical decisions. Quantitative analysis of digital images requires detection and segmentation of the borders of the object of interest. In medical images, segmentation has traditionally been done by human experts. Even with the aid of image processing software (computer-assisted segmentation tools), manual segmentation of 2D and 3D CT images is tedious, time-consuming, and thus impractical, especially in cases where a large number of objects must be specified. Substantial computational and storage requirements become especially acute when object orientation and scale have to be considered. Therefore automated or semi-automated segmentation techniques are essential if these software applications are ever to gain widespread clinical use. The main purpose of this work is to analyze segmentation techniques for the definition of anatomical structures under telemedical systems.

## 1. Introduction

Access to medical care is sometimes very difficult to be reached from people living in rural and underserved areas. The implementation of systems support different telehealth technologies as well as on evidence-based medicine. A fundamental change in the management and delivery of healthcare, by leveraging telehealth applications and solutions, is crucial to get the most cost-effective and efficient use out of available healthcare resources. It is therefore crucial to develop a general information exchanged e-medical system to enable its users to perform online and offline medical consultations. This means by consultation of additional medical experts, medical diagnosis can be stated more precisely, and certified. During the medical diagnosis, image analysis techniques combined with doctor's opinions could be useful for final medical decisions.

Telehealth is the exchange of health information and the provision of health care services through electronic information and communications technology, where participants are separated by geographic, time, social and cultural barriers [1]. It represents a wide range of variables including clinical application, characteristics of the information being transmitted, temporal relationships of data transfer, and the organizational context [1]. In quality assessment studies on information systems effectiveness, data quality, the ISO and standards and issues related to the quality of medical care exist already.

Over the past years, simulator-assisted training in healthcare has become increasingly important. Simulation refers to the artificial generation, representation, and interaction of a complex-real-world process. In the medical area the trainee has the ability to realistically learn a number of skills without the physical presence of a real patient. The benefits are availability and evaluation of different medical cases; clinical human performance and mastering complex or even critical situations offers a safe study environment in absence of the physical patient, especially in cases where the distance is a crucial parameter for the diagnosis and treatment. Because there is a close relation between telemedicine and medical imaging, it is essential to develop e-medical systems appropriate to provide information for the anatomical structure of the human body.

Moreover the online-mode gives the opportunity to communicate over far distances with a given partner in real time. In this case both partners look at the same image material and through text messages (chat window) and transfer mouse actions to the remote PC, where they can discuss interactively over a medical case.

Methods for providing that kind of information are based on segmentation techniques. Methods for performing segmentations vary widely depending on the specific application, imaging modality, and other factors like treatment (e.g., cancer treatment, image-guided surgery, and invasive techniques) or diagnostic imaging [2–4].

In this work we will present segmentation techniques that improve the time and interaction one needs to define one or more structures. This methodology involves interpolation methods for the volume definition (manual and semiautomatic) and methods for the semiautomatic organ shape extraction. Once an accurate segmentation is obtained, this information may be used by the doctors to compare the volume and morphology characteristics of each region against known anatomical norms, other regions in the same image set, and corresponding regions in related image sets.

## 2. Motivation

Telemedicine, and recently e-health, are the names of telecommunication tools, which are able to improve the equitable access to healthcare. The ability of telemedicine to facilitate medical care, irrespective of distance and availability of medical specialists on site, makes it attractive to the healthcare sector. Telemedicine allows better utilization of scarce medical personnel and resources. Telemedicine promises to enhance continued on-the-job medical training of doctors, nurses, and other healthcare practitioners in rural areas, and this perspective is extremely useful for all developing countries.

Telemedicine is defined as the practice of medicine at a distance, where a messenger carrying over a medical advice from a distant expert to the bed of a patient. Medical care can be provided through these systems almost anywhere at anytime. The physicians and health care organizations are therefore able to guarantee high-quality care, without the patient having to travel and being carried over long distances.

Effectiveness of the telemedical systems could be seen especially in demanding diagnostics (cancer disease) where distance is an important factor in patient treatment (radiotherapy). Radiotherapy is one of the most effective forms of cancer treatment, undergone by roughly a third of all cancer patients. Once cancer has been diagnosed, patients generally have three fundamental treatment alternatives: surgery, radiotherapy, or chemotherapy. None of these options is particularly comfortable, and they each have positive and negative aspects. Radiotherapy is the only choice in certain cases, such as fast-growing tumors, in the postoperative treatment of breast or lung cancer, some prostate types and many others, where radiotherapy is often used to prevent the regrowth of malignant tissue.

The scope is on the one hand the virtualization of the complete treatment planning procedure (to deal with the digital patient data rather than the real patient), on the other hand to make the virtual patient available on different sites than those of its physical location and therefore allow telematics cooperation of radiooncology centers. The first fact will mainly increase efficiency of personnel and accuracy (quality) of treatment, while the second will mainly reduce costs by allowing virtual sharing of extremely expensive devices as well as remote expert assistance/service. Although system demonstration will be based on a worldwide commercially available radiotherapy planning system which will extend its efficiency by adding telematics components, mainly based on generally available networks (Internet, ISDN, phone lines).

The main efforts will be entirely focused on developing the innovative telematics tasks and implementing these on existing professional and commercially available technology. Due to the availability of telematics components expensive devices such as CT (Computed Tomography) scanners will become virtually available to hospitals without such devices physically present. In addition, remote experts can generate the treatment plan of a patient and/or validate plans of their colleagues on-line (consultation conference) or off-line (desktop system).

Powell III [5] states that telemedicine programs can help patients in remote areas being cared at their duty station, when they might otherwise have traveled for care. As a result there is an increase in appropriateness of consultation request, improved access to care, enhanced provider education, and higher customer satisfaction.

## 3. Telemedical Platform

To generate views from different anatomical structures contained in CT data sets or to perform treatment planning techniques, it is essential (1) to extract the corresponding data from the image and (2) to accurately define the target volumes as well as the organs at risk for the treatment outcome; both methods can be done via segmentation process.

The first fact will mainly increase efficiency of personnel and accuracy (quality) of treatment, while the second will mainly reduce costs by allowing virtual sharing of extremely expensive devices as well as remote expert assistance/service. The main efforts will be entirely focused on developing the innovative telematics tasks and implementing these on existing professional and commercially available technology.

**Figure 1:**
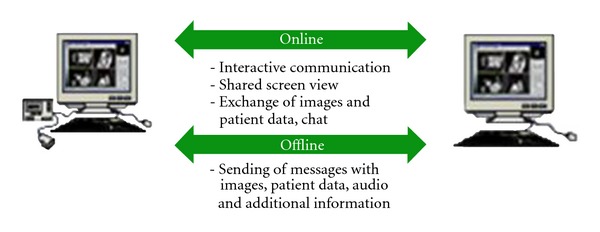
Online and offline telecollaboration.

In order to make possible the data transference, a telemedical platform must be implemented. Aim of the platform is the collaboration between doctors and/or citizens located in countries with different health care systems, different technology infrastructure, and different needs, in order to enhance the quality of health care services and the equal access to the health care systems. Through the platform will be possible the efficient handling of patients with chronic diseases, the early response to emergency incidents, and the reduction of human lives losses. Based on the system platform users are able to perform online and offline medical consultations. In addition to the given possibilities telemedical systems offer the following features: (1) sending and receiving of multimedia messages with text, image material, and audiomessages, over Internet, ISDN and analog phone line in offline and online mode. (2) Graphical and textual annotations and pictograms can be added to the images. (3) In the online mode interactive communication via usage of a chat window, mouse, exchanged images, and a consolidated screen view is possible. Support of the operation in a telemedicine center and consultation clients through a client-server database all image of the patient and message-information will be stored constantly. The principle of the online teleconsultations is to bring two geographically distanced doctors together in an online session. During that session, both doctors will have the same view on the images and can communicate through additional communication features like chat, annotations, and so forth. In that way, expert knowledge and advice is provided to doctors in rural and crisis situation areas, or simply to any doctor in the field, who needs help in diagnosing a clinical case and do not want or is not able to send the patient to the expert physically (Figure 1). Extended examples of telemedical systems are described and illustrated in [6, 7].

**Figure 2:**
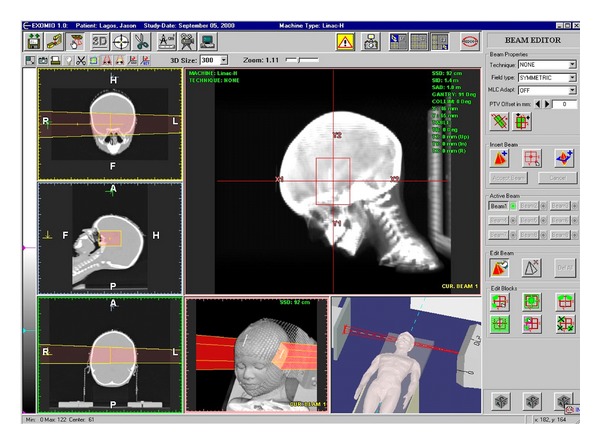
The six-window layout of the system. On the left side the slices windows, the middle lower window is the OEV, the lower right the Room View, and the upper right hosts the BEV [21].

These systems have the ability to generate 2D and 3D images using only the original CT data of the patient. The 2D images displayed are the original axial CT, MR, or PET. Multiplanar reconstructions (MPR) can be generated in real time in the orthogonal, coronal, and sagittal directions, and any oblique direction. The 3D reconstructed images are a must in CT-Sim systems in order to simulate the patient anatomy and the images generated from the real simulator or the treatment machine. The first layout contains four windows and the displayed images are the Beam's Eye View (BEV), the Observer's Eye View (OEV) and the Room View (RM), together with the axial slices. The second layout is composed again of four windows, containing the three orthogonal slice directions and the OEV. This layout is ideal for navigation through the CT volume, for volume delineation and to observe complex radiation field arrangements. The last layout contains every image described above. This layout has six windows emphasizing the size of the BEV image, since the physicians feel comfortable working with this image. In both rendering views, OEV and BEV, the volume orientation is controlled using a mouse track ball. The important RTP imaging is the dynamic X-ray image on the Simulator monitor, which is generated from the viewpoint of the beam source, called Beam's Eye View (BEV) image, see the BEV view direction in Figure 5. In the system the corresponding window, called the BEV window, displays the interactive DRR images. Through the BEV window, the physicians can investigate the patient anatomy in the DRR image as they observe it on the Simulator monitor. Observer's Eye View (OEV) window visualizes the patient from the room's eye view, which is the same view as the physicians observe the patient in the Simulator room, see the OEV view direction in Figure 5. As we explained before, all of the images in both BEV and OEV windows are generated with the patient CT scan by the direct volume rendering (Figure 2).

The system can use large amounts of data, from 40 up to 300 slices, in order to produce high-quality anatomical images. It enables reading of CT data, acquired not only with a single slice CT scanner (such as a Siemens SOMATOM Plus 4), but also multislice data ranges, acquired in this study with four slices (SOMATOM Volume Zoom). It can be connected via network directly to any CT scanner that supports DICOM protocol. The system allows the adaptation to any LINAC configuration through a machine configuration module that is designed according to the international standard for radiotherapy equipment (IEC 1217). This configuration involves limitation on movements of the mechanical component and description of the component's dimensions (e.g., multileaf collimator, or MLC).

**Figure 3:**
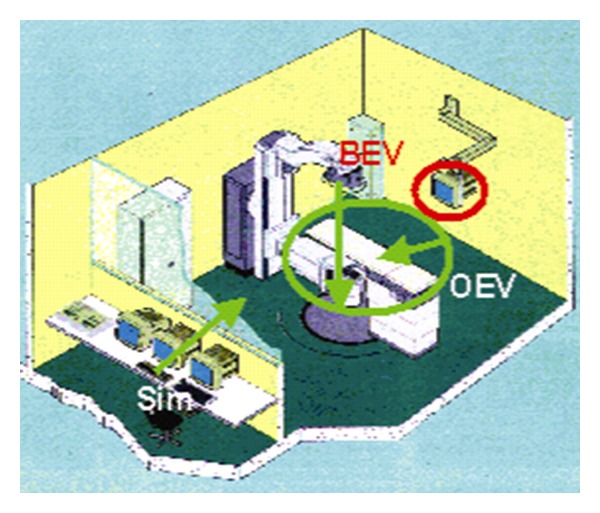
Simulation System.

Instead of the presence of the physical patient's body, a virtual patient's body as well as the VS machine is displayed, which is called the Simulator Window. Through the Simulator window, the physicians can investigate the movements of the machine as they observe it through the lead glass in their Simulator operation room; see the Sim view direction in Figure 3.

## 4. Radiotherapy Treatment Planning (RTP)

**Figure 4:**
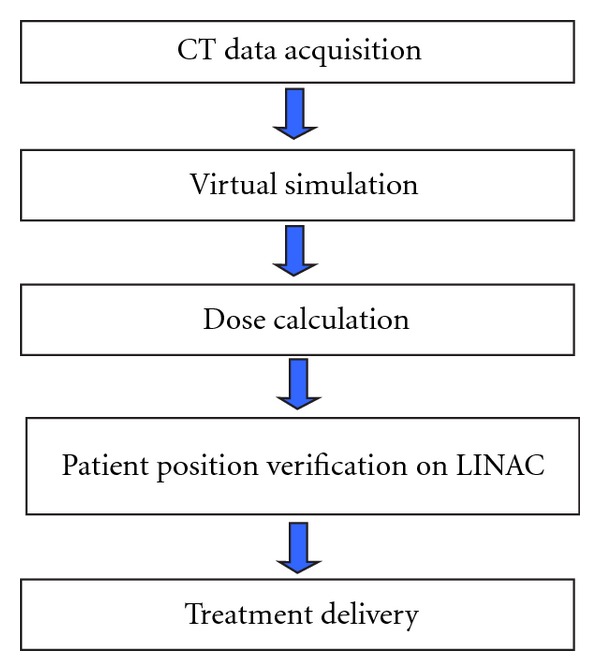
Current clinical routine for external beam treatment delivery [21].

The effect of the radiotherapy treatment is based on the precise delivery of high irradiation dose on the tumor site without damaging the surrounding healthy tissues. Therefore patient positioning, target volume definition, and irradiation field placement are very critical steps while planning the irradiation process. One of the significant technological advances in radiotherapy in the past 20 years is the implementation of CT or Virtual Simulators (VS), in the clinical routine. Sherouse et al. in 1987 [8] first proposed the concept, often termed CT-Sim to distinguish it from Sim-CT where a simulator is modified for CT use and by the late 1990s several designs and clinical assessments of CT virtual simulators have been reported [8–15]. Using VS, the clinical routine is modified accordingly (Figure 4) [16, 17].

1. Collect patient's CT data including attached aluminium markers.
2. Transfer CT data to VS. The physician defines the tumour volume and the organs at risk and she/he will place the necessary fields relative to the tumour volume.
3. The simulation plan and the CT data are transferred via DICOM (Digital image and Communication in Medicine) server to the TPS for dose calculation and final treatment plan optimization.
4. Verify patient position on LINAC before irradiation.
5. Perform treatment on the treatment machine (Linear Accelerator or LINAC).

During the images' transferring in teleradiology important factor for effective diagnosis is the exchanging time. Crucial involvement to this achieved has the volume rendering process. Volume rendering is the technique according to which a scalar field of data with discrete values, volume data, is selectively sampled in order to generation a useful image in relation to the sampled values. A volume data set is typically a set of discrete samples of one or more physical properties in a limited object space.

A volume data set is typically a set of discrete samples of one or more physical properties in a limited object space, *V*(*x*), *x* ∈ *R*_*n*_, in which {*x*} is a set of sampling points; *n* is the dimension of the sampling space, usually *n* = 3, that is, 3D volume data; *V* represents the sampling values, it can be a single-valued or multivalued function. According to the distribution of sampling points (i.e., the structure of *x*), volume data sets are classified into structured and unstructured data sets. In medical imaging, volume data is usually a structured data set, typically organised as a stack of slices; *V* can be single valued (e.g., the CT Hounsfield value) or multivalued (e.g., density, T1, T2 in MRI). The resulting data structure is an orthogonal 3D array of voxels, each representing a sampling value.

**Figure 5:**
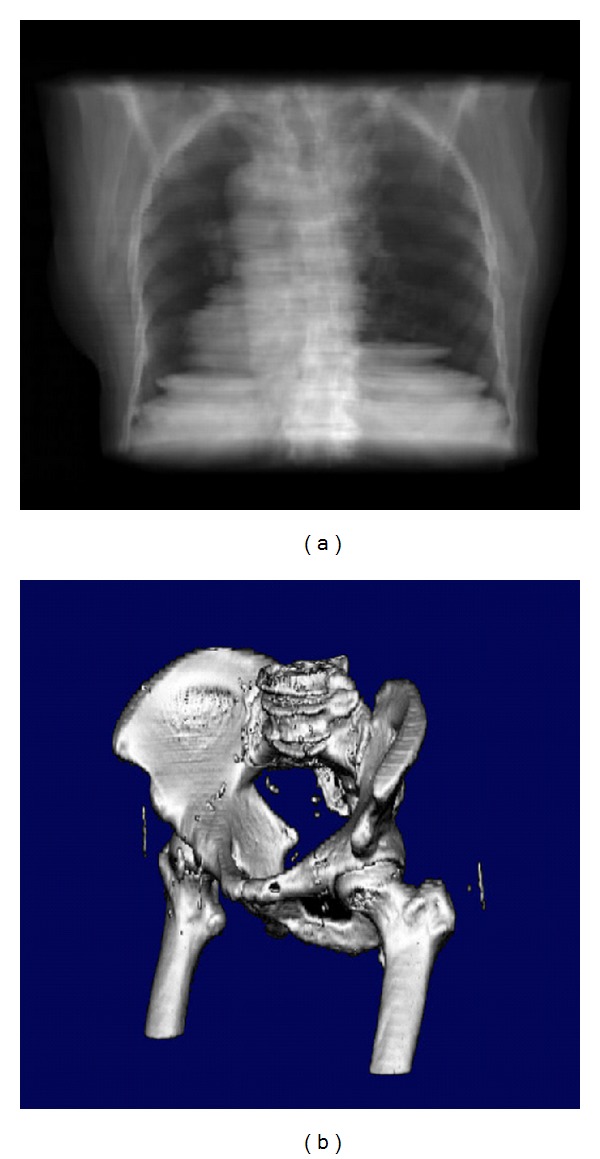
(a) Reconstruction of CT volume using DRR; (b) Reconstruction using direct volume surface using direct gradient volume estimation [21, 28, 33].

The rendering methods which are proposed in this work are based on the work of [18]. (1) Transparent mode: Digitally Reconstructed Radiographies (DRR) with general energy exponential equation:

(1) $$I\left(s\right)=I_{0}\exp\left(-\overset{}{\underset{D}{\int }}K_{\lambda }\left(t\right)dt\right),$$

where *K*_*λ*_ is the attenuation coefficient and *λ* is the wavelength in distance *D* [18]; (2) surface reconstruction mode: isovalue, gradient [19] and semitransparent with general energy form:

(2) $$I\left(s\right)=\overset{}{\underset{D}{\int }}C\left(t\right)\alpha \left(t\right)T\left(t\right)dt,$$

where *C*(*t*) is the shading intensity at point *t*, *α* is the opacity, and *T*(*t*) is the accumulated transparency from 0 to point *t* in distance *D*. DRR images generated from CT digital volumes are often called using the term digitally reconstructed radiographs (DRRs) (Figure 5). 

To reconstruct a DRR the digital CT data of the patient are used. Each voxel acquired using CT has a value that is called the Hounsfield unit upon the name of the father CT. The HU can be estimated using the following formula (3):

(3) $$\text{HU}=\frac{\left(\rho_{\mu }-\rho_{\text{water}}\right)}{\rho_{\text{water}}}*1000,$$

where *ρ*_*μ*_, *ρ*_water_ are the attenuation coefficient of the material for the specific voxel and the water, respectively. When on the above equation one replaces the *ρ*_*m*_ = *ρ*_water_, then will receive a HU_water_ = 0. In addition the air as material corresponds to −1000HU, since *ρ*_air_ = 0. The Hounsfield units have no upper limit but usually for medical scanners a range between −1024 to +3071 is provided. Apparently 4096 different HU values are provided and therefore to illustrate the complete range of the HU on a volume 12-bit voxels are required.

Probably the most common methods for reconstructing surfaces directly from voxels are the gradient surface and isosurface model. This rendering model is widely used in almost all medical data sets to render the surface detected by the gradient operator. Levoy [20] presented this concept, which is effectively used in most medical imaging applications for the last decade. Depending on the data set size and on the size of the resulting 3D image, which influence the number of rays, a 3D view image can be calculated almost in real time (0.8 sec to 2.3 sec).

**Figure 6:**
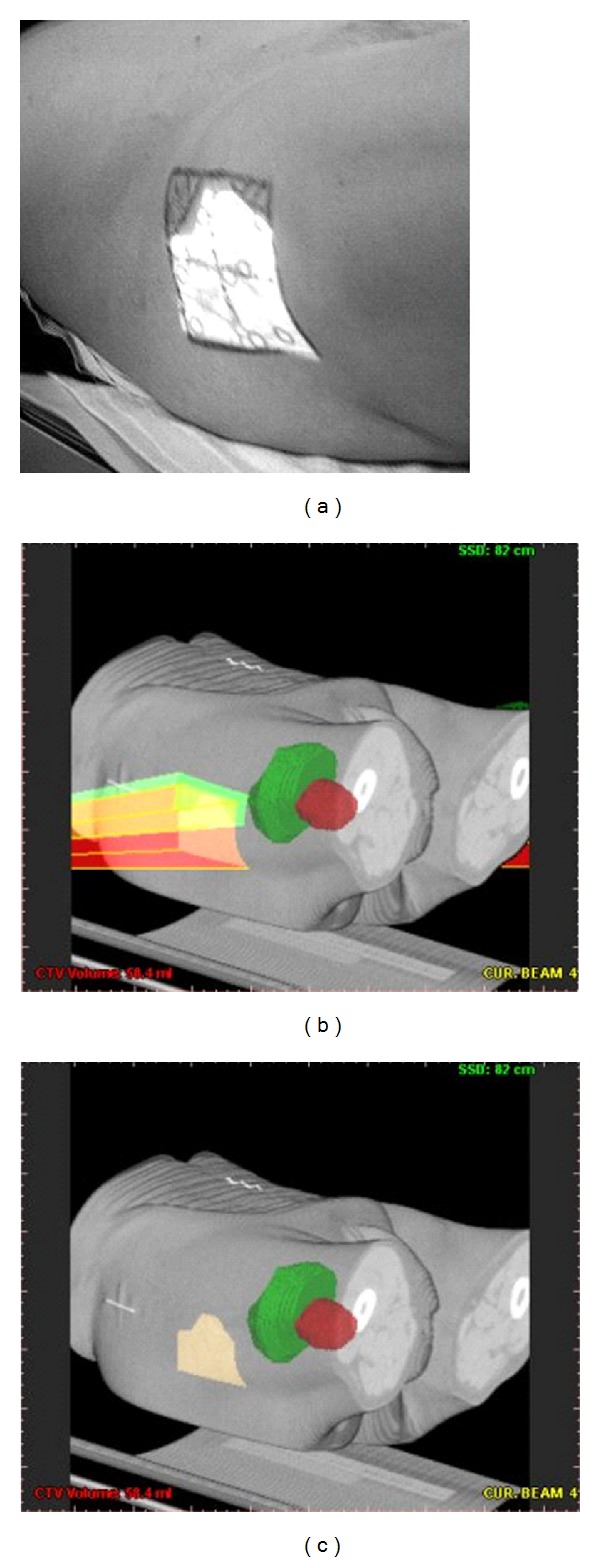
On (a) the light field projection, delineated from the block shape. In (b) the 3D reconstruction of the beam and block object. On (c), the delineated virtual light field projection [21].

Before applying any visualization routine the beam and block polygon are rotated and translated so as to achieve the appropriate orientation relative to the CT data. The common visualization of beam, block polygons, and the CT data is achieved using a 3D scan-line conversion algorithm. Each polygon's plane is scanned from side to side. For the 3D visualization of the polygon, the depth value of the point plane is compared with the depth information of the surface Z-buffer that is calculated by the volume-rendering pipeline. If the depth value of the point plane is closer to the spectator than the calculated surface position, then the original pixel value is mixed with the point plane color value (Figure 6). If *Z*_*V*_(*i*, *j*) is the function during the volume rendering process. If one compares the current edge Z-depth *Z*_*P*_(*i*, *j*) with the value of the image Z-buffer then could make the following two assumptions [21].

- If *Z*_*P*_(*i*, *j*) < *Z*_*V*_(*i*, *j*) then the current polygon edge is behind the volume and is hidden from the viewer. In this case no value for the light mask is accumulated.
- If *Z*_*P*_(*i*, *j*) > *Z*_*V*_(*i*, *j*) then the current polygon edge is in the front of the volume and is visible from the viewer. In this case one value for the light mask is accumulated.

## 5. Segmentation Techniques

Image segmentation is an essential step for many advanced imaging applications. Accurate segmentation is required for volume determination, 3D rendering, radiation therapy, and surgery planning. Traditionally, image segmentation denotes the technique of extraction of image entities or structures (regions or objects), so that the outlines of these structures will coincide as accurately as possible with the physical 2D object outlines [22, 23]. An object region is a set of image pixels, which are similar with respect to a homogeneity criterion such as texture.

A problem that occurs is that images either over-estimated, with objects divided into parts or images are incorrectly segmented, with or without more objects segmented as one object. An obvious direction is the use of the manual methods. In this work we will present an effective semiautomatic method, based on the boundary tracking technique [24], which improves the time when one or more structures are in use. The implemented algorithms can segment within a few seconds the complete volume of specific organs, for example, lungs, skin, and spine. The only interaction of the user is to select the starting point in the region of interest and the algorithm will track the object boundaries in 3 dimensions. The segmentation process might involve complicate structures and in this case usually only an expert can perform the task of the identification manually on a slice-by-slice base. Humans can perform this task using complex analysis of shape, intensity, position, texture, and proximity to surrounding structures. Of course, all these features are differently qualified depending on the experience of the user. To perform a similar procedure automatically using a computer since today has been proved a very difficult task. In other cases where simpler anatomical regions with a very distinguishable shape must be identified an algorithm can perform this task. Currently there are only dedicated algorithms for different regions of the human body. To generate a “complete,” with a general meaning of the term, segmentation application numerous tools and algorithms must be combined [25].

Image segmentation approaches may be performed in one of three ways: (1) completely manual: an operator with proper training draws the required boundaries manually, using a mouse or other pointing devices. This requires expert knowledge of the operator and skills in drawing boundaries with available tools. Manual drawing of boundaries is difficult and time consuming. It will pose a serious problem when the size of data sets grows, (2) fully automatic: the current available techniques, such as thresholding or region growing are not sophisticated and robust enough for our application. The use of these techniques is therefore restricted to simple boundaries and requires high image quality, and (3) semiautomated: this method uses boundary finding results generated by sophisticated automatic algorithms as initial guess, and then allows an operator to edit the required boundaries manually. This method still requires expert knowledge of the operator, but reduces the time and effort needed. The differences in these types of interaction are the amount of time and effort required, as well as the amount of training required by an operator. Even automated segmentation methods typically require some interaction for specifying initial parameters that can significantly affect performance.

**Figure 7:**
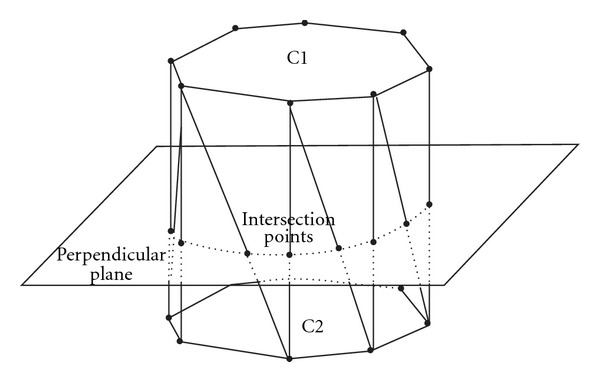
On the left side a simple case where an interpolated contour is created from the plane intersection with the triangles [33].

*Linear Interpolation* is one of the simplest methods where linear interpolation between contours is the first approach used to provide an acceleration tool for the manual contouring. The mechanism of the linear interpolation is applied when between the key contours at least one slice exists. To perform the linear interpolation we create triangles between the contour points of the key contours as described in Sakas [17]. For this operation both contour's points must be rotated towards the same rotation direction. The interpolated contour points are created after calculating the intersection of each triangle side with the intermediate slice (Figure 7). After the definition of the organ boundaries for a given 2D shape, a surface reconstruction of the 3D data defining anatomical structures must be defined. 

**Figure 8:**
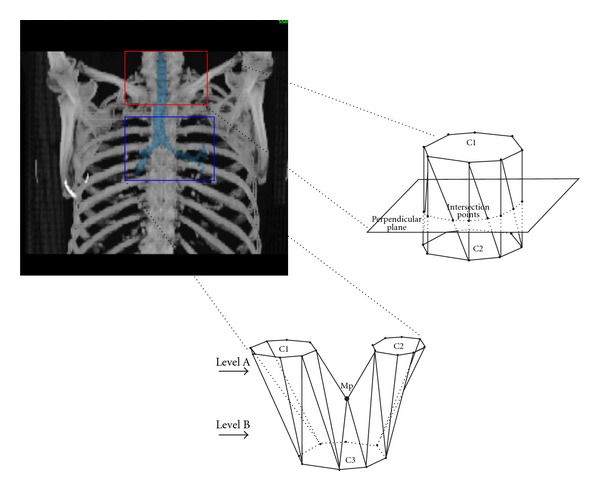
Contour samples defined according to trachea and bronchus shape. 3D segmentation and linear and bisection interpolation using surface rendering technique.

Linear interpolation between key slices is a very common approach used in RTP process to accelerate target volume definition. To overcome the problem with local discontinuities when one contour every time is considered, a linear interpolation technique based on surface rendering is applied using information from the triangulation of the 3D shape. Surface rendering is a process in which apparent surfaces are produced from the data volume and an image of the extracted surfaces is suitably visualized. Surface contours are modelled with bidimensional primitives, as triangles or polygons, in order to represent a 3D object. The basic idea is to extract a surface from the 3D data volume as a collection of adjacent polygons and to visualize the extracted surface by appropriate algorithms. The triangulation approach is described as in Figure 7: contours defined on two adjacent slices are divided into the same number of equiangular sectors. The points corresponding to sector neighbours are connected by obtaining a series of triangles that will define the surface. The marching-cube algorithm works on a 3D mask that defines the object of interest obtained by a threshold-based or a more complex segmentation algorithm (Figure 8). 

*Semiautomatic segmentations methods* combine the benefit of bath manual and automatic segmentation techniques. By supplying initial information about the region of interest, the user may guide an otherwise automatic segmentation procedure. Any remaining errors introduced by automatic segmentation methods may be corrected by manual editing. In this work, a boundary tracking algorithm [26–30] was implemented for the segmentation part.

**Figure 9:**
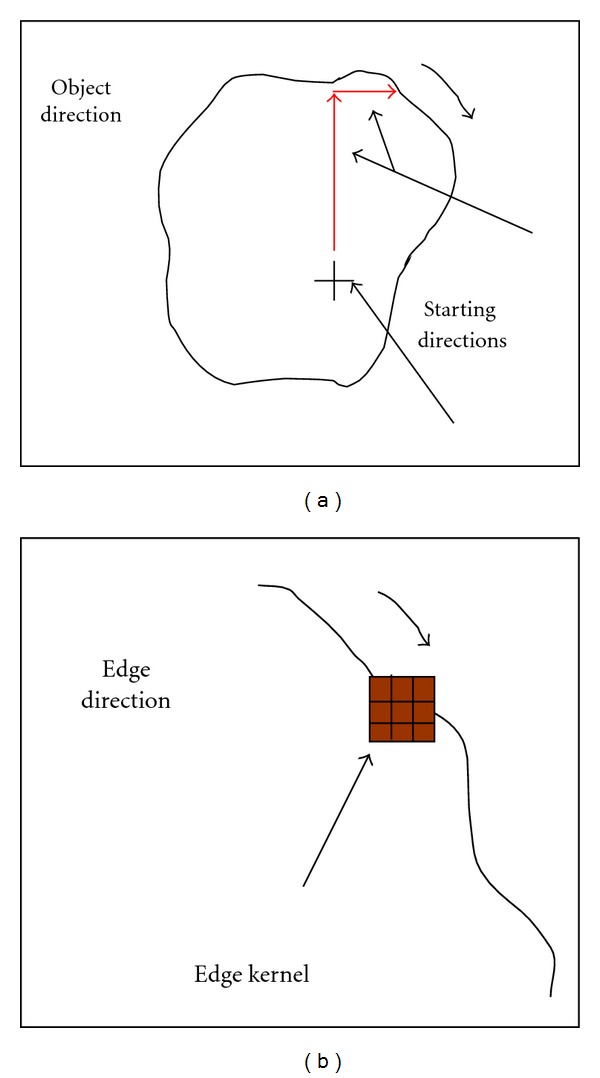
(a) Boundary tracking method. (b) Contour filtering.

Boundary tracking technique (BT) is a segmentation method that given one point along a region's boundary follows the boundary around the region until it returns to the original point. Assuming a constant boundary, this allows for considerable variation within the region without any effect on the segmentation. The advantage of the approach is that no assumptions are needed to be made a priori about the boundary shape, which may vary from a straight line too much more complex shapes which are difficult to parameterize. First let us assume that the image with regions is either binary or that regions have been labeled using a specific threshold (white for the organ of interest, black for the background). The algorithm starts with an initial point. Different starting directions are defined until a sharp edge was found. Each boundary was tracked, keeping to the right part consistently. The tracking procedure stops only when all the boundary regions have been scanned. The algorithm traces the edges with detail providing high accuracy to the description of the contour shape (Figure 9(a)).

Image-smoothing techniques are used in image processing to reduce noise. Usually in medical imaging the noise is distributed statistically and it exists in high frequencies; therefore, it can be stated that image smoothing filters are low-pass filters. The drawback of applying a smoothing filter is the simultaneous reduction of useful information, mainly detail features, which also exist in high frequencies.

The basis for the filtering operation is the convolution operator, where a *K* × *K* matrix known as the kernel or mask is introduced by a general form:

(4) $$\begin{array}{c} w_{ij}=\left[\begin{array}{ccccc} w_{11} & w_{12} & \ldots & \ldots & w_{1k}\\ w_{21} & w_{22} & \ldots & \ldots & w_{2k}\\ \vdots & \vdots & \ldots & \ldots & \vdots \\ \vdots & \vdots & \ldots & \ldots & \vdots \\ w_{k1} & w_{k2} & \ldots & \ldots & w_{kk} \end{array}\right],\\ f_{ij}=\left[\begin{array}{ccccc} f_{11} & f_{12} & \ldots & \ldots & f_{1k}\\ f_{21} & f_{22} & \ldots & \ldots & f_{2k}\\ \vdots & \vdots & \ldots & \ldots & \vdots \\ \vdots & \vdots & \ldots & \ldots & \vdots \\ f_{k1} & f_{k2} & \ldots & \ldots & f_{kk} \end{array}\right], \end{array}$$

where **f** is the original image. The convolution process is defined by **g** = **w** ⊗ **f**. The convolution filter with the mask can also be used to find contours inside an image; especially in volumetric and surface processing. It is the first step of contouring detection, where the kernel isolates the part of the image where edges appeared (large steps, rapidly changes). After segmenting the regions of interest, a 3 × 3 Sobel edge detection operator (Figure 9(b)) is used to improve the appearance of the contours.

In the analysis of the objects in images it is essential that we can distinguish between the objects of interest and “the rest.” This latter group is also referred to as the background. The techniques that are used to find the objects of interest are usually referred to as segmentation techniques—segmenting the foreground from background. Image thresholding is a technique for converting a grayscale or color image to a binary image based upon a threshold value. If a pixel in the image has intensity less than the threshold value, the corresponding pixel in the resultant image is set to white. Otherwise, if the pixel is greater than or equal to the threshold intensity, the resulting pixel is set to black. For that purpose, a low-pass filter was used based on the rectangular window or box function based on the rule:

(5) $$I\left(i,j\right)=\{\begin{array}{cc} 1, & \text{if}\;I\left(i,j\right)\geq T\Rightarrow \text{object},\\ 0, & \text{otherwise}\Rightarrow \text{background}, \end{array}$$

where *I*(*i*, *j*) is image matrix and *T* is the appropriate threshold. The output is the label “object” or “background” which, due to its dichotomous nature, can be represented as a Boolean variable “1” or “0.” The central question in thresholding then becomes how do we choose the threshold? While there is no universal procedure for threshold selection that is guaranteed to work on all images, there are a variety of alternatives. For this case a histogram-derived threshold was chosen based on the image histogram combined with Hounsfield unit tissue values. To overcome the problem with the increasingly high number of points, that are reconstructing the contour, a point decimation algorithm (or sequence sampling algorithm) was used to reduce the number of points without loss of their general presentation. If a sequence of numbers was given, *S* = {*s*_1_, *s*_2_, *s*_3_,…, *s*_*i*−1,_ *s*_*i*,_ *s*_*i*+1,_ …, *s*_*n*_}, then the main idea is to construct another sequence S*, where *k*%-values would be removed from the original sequence. Summarizing the above procedures, the pseudocode for the segmentation technique is presented in Algorithm 1.

The main drawback of the method is that it is a binary approach and hence is very sensitive to gray value variations. If the threshold value is not selected properly then the system will fail to detect the appropriate organ shape. Most of the inaccuracies of the segmentation method require the user intervention to optimize the result. To overcome the limitation we calculate a secondary opacity volume from the original CT data based on the well-known approach from [31], that is very often used to visualize surfaces from scalar volume data in volume rendering.

For the lungs segmentation, two regions would be investigated: (a) concave and (b) convex. For the convex regions firstly different starting directions was defined. The algorithm requires an initial point to start the tracing of the edge of the object under investigation. The initial point travels to the vertical or horizontal direction until the edge of the investigated object is reached. Then the algorithm will start to exam the surrounding pixel of that edge and check whether they belong to the current edge or not. For the concave regions a different approach was achieved implementing a two moving position methods based on where the starting point appears: (i) outside point and (ii) inside point. The algorithm uses a constant threshold selection with levels 20 to 70 Hounsfield values.

**Outside Point**

Considering the case where the object is *concave* and a *starting point* was given, extended experiments indicate false segmentation alarms, when the starting point remains at the same position for all the slices; to overcome this, a region growing technique (grey color) was applied to find the first white region inside the organ; a region growing approach (green color) was used, to avoid undesired results in case the point position was moved at the edge of the region.

**Figure 10:**
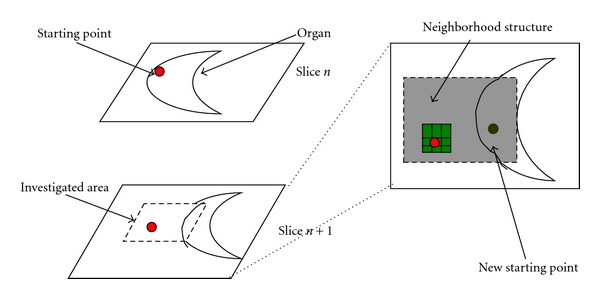
Moving position process (outside position).

The region-growing (RG) algorithm is used very often in the region-based segmentation techniques and has been successfully incorporated into several medical imaging applications for the extraction of organ volumes. In this work we follow a simple design principle since the region boundaries that must be detected have low complexity and high contrast differences with the surrounding tissues. The RG algorithm is initiated with a seed voxel selected manually, which is known to be a part of the voxel of interest, room air or skin surface. If the difference in grey level is between the voxel under investigation and its six adjacent voxels are less than a given threshold, then the current voxel is merged with the growing region. This process is repeated recursively to grow the object until not more voxels can be found. The starting point changes and the boundary tracking technique continue as before (Figure 10).

**Inside Point**

**Figure 11:**
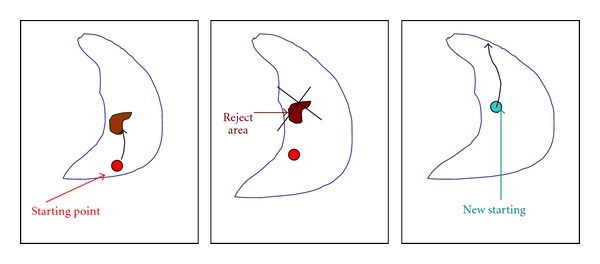
Moving position process (inside position).

Consider now the case where the starting point is inside the lung region and close to the area of high tissue (small white spots). In this case using the original method, the tracking technique probably will spot (for some slices) the white areas avoiding the bigger lung region. Extended experimental results suggest that these regions have maximum area close to 6 cm^2^: if the object is smaller than 6 cm^2^ then it is removed and the process continues until a larger region corresponds to the overall lung area is found. With this restriction the method avoids to segment areas that are meaningless (Figure 11).

**Figure 12:**
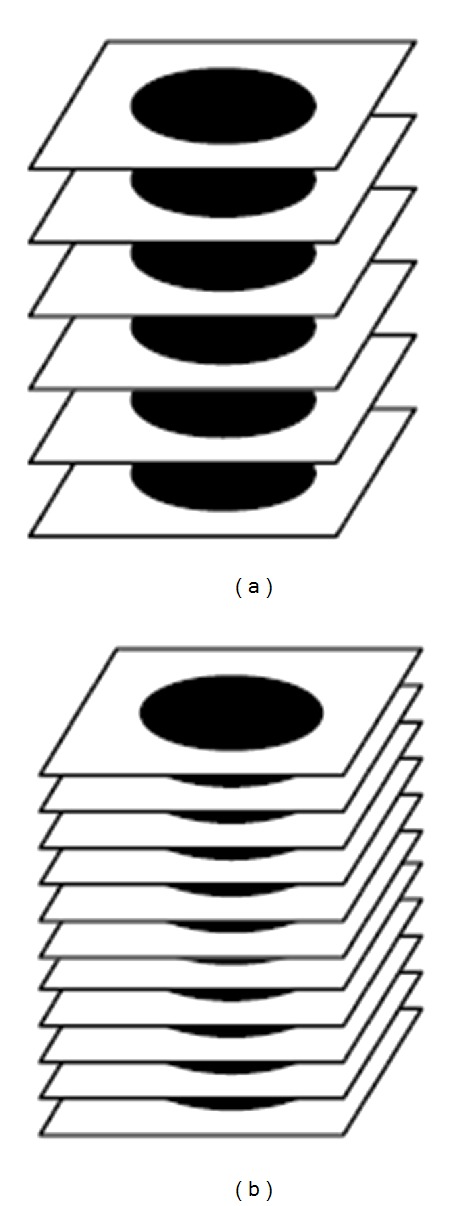
Slice interpolation.

For visualizing volumetric medical data, sequences of 2D images are piled up to recreate the three-dimensional structure (Figure 12(a)). Usually, in this step linear interpolation of adjacent slices is needed for the generation of new slices (Figure 12(b)), since one of the problems resulting from image acquisition is the space between slices. This problem occurs because the sampling interval between slices is normally greater than the generated image resolution, and then the volume voxels are not cubic. After interpolation this size distortion is corrected, so that the visualization algorithm could generate correct proportion projections.

Assuming a simple 1D example of sampling points linear interpolation offers first-degree connectivity among the sampling points, passing a straight line through every two consecutive points of the input signal. In the spatial domain, linear interpolation (LN) is equivalent to convolving the sampled data with the following interpolation kernel (6):

(6) $$h\left(x\right)=\{\begin{array}{cc} 1-\left|x\right|, & \text{when}\;0\leq \left|x\right|\leq 1,\\ 0, & \text{when}\;1\leq \left|x\right|. \end{array}$$

The above interpolation kernel corresponds to a reasonably good low-pass filter in the frequency domain [32]. In practice we can define the linear interpolation between two points *p*_0_, *p*_1_ in one dimension as follows (7):

(7) $$p_{x}=\left(1-x\right)p_{0}+xp_{1}\Rightarrow p_{x}=p_{0}+x\left(p_{1}-p_{0}\right).$$

In two dimensions the interpolation scheme will involve four data points' *p*_00_, *p*_01_, *p*_10_, and *p*_11_. The interpolation can be formulated using the following formula (8):

(8) $$\begin{array}{cc} p_{xy} & =\left(1-x\right)\left(1-y\right)p_{00}+\left(1-x\right)yp_{01}\\ & \;+x\left(1-y\right)p_{10}+xyp_{11}. \end{array}$$

This interpolation method is known as *bilinear *interpolation and is commonly applied on image interpolation for example, when magnifying raster images.

**Figure 13:**
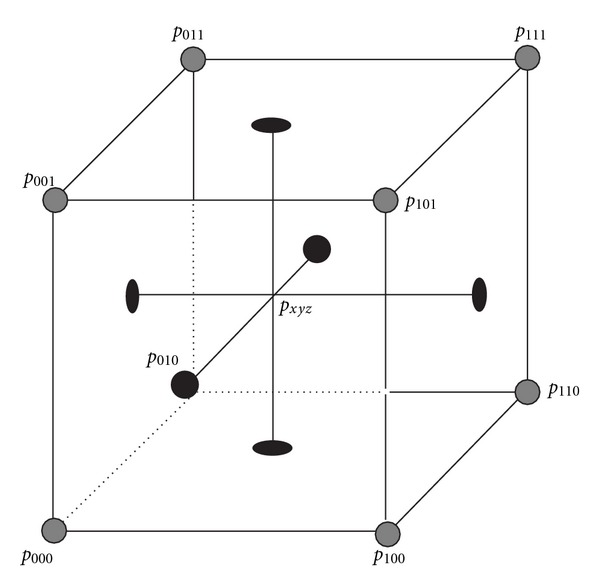
The geometry of a unit cube with the data points lying on the edges and the interpolated value in position (*x*, *y*, *z*).

In volumetric data linear interpolation is known as tri-linear interpolation and estimates the value of the result voxel considering the neighbourhood of eight (8) voxels (*p*_000_, *p*_001_, *p*_011_, *p*_010_, *p*_100_, *p*_110_, *p*_101_, and *p*_111_) (Figure 13) with estimation formula for *p*_*xyz*_ (9):

(9) $$\begin{array}{cc} p_{xyz} & =\left(1-x\right)\left(1-y\right)\left(1-z\right)p_{000}+x\left(1-y\right)\left(1-z\right)p_{100}\\ & \;+xy\left(1-z\right)p_{111}+\left(1-x\right)y\left(1-z\right)p_{010}\\ & \;+\left(1-x\right)\left(1-y\right)zp_{001}+x\left(1-y\right)zp_{101}\\ & \;+\left(1-x\right)yzp_{011}+xyzp_{111}. \end{array}$$

**Figure 14:**
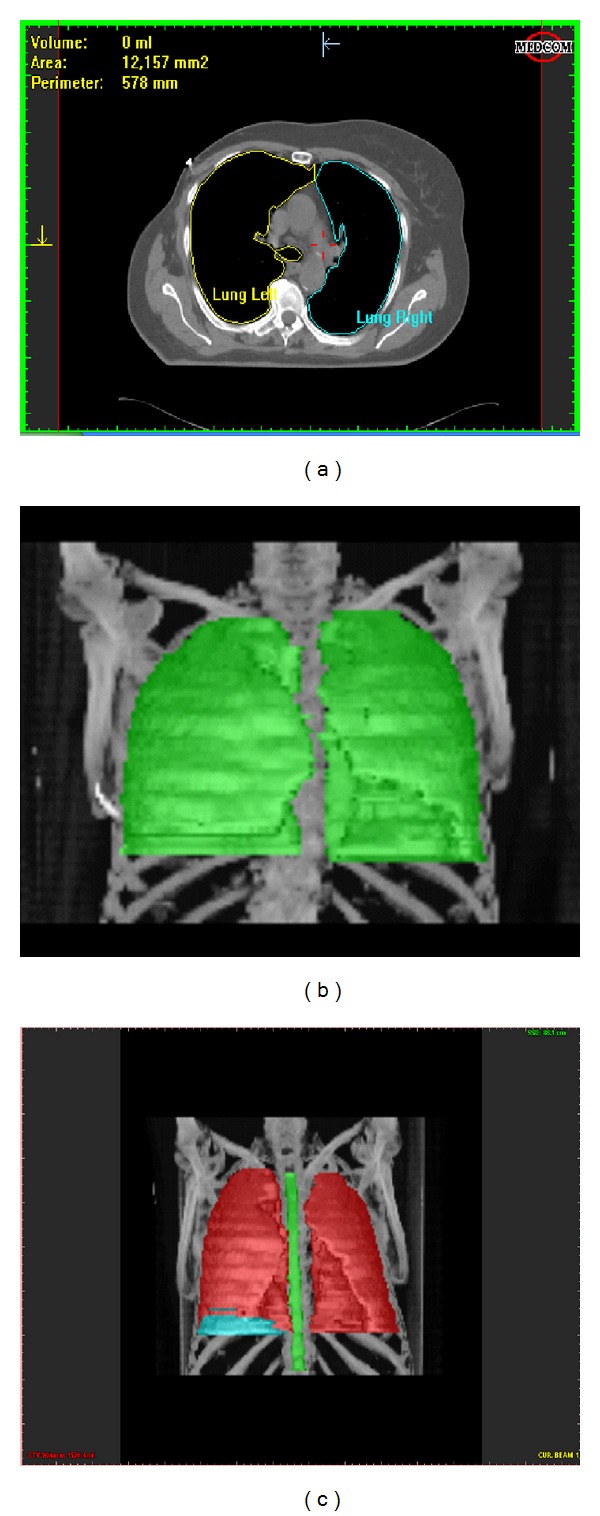
Segmentation of different anatomical structures. (a) 2D segmentation of the left and right lung; (b) 3D segmentation of the left and right lung; (c) 3D segmentation of the lungs, spine, and tumor.

Figure 14 illustrates different 2D and 3D reconstruction examples of segmented structures.

**Figure 15:**
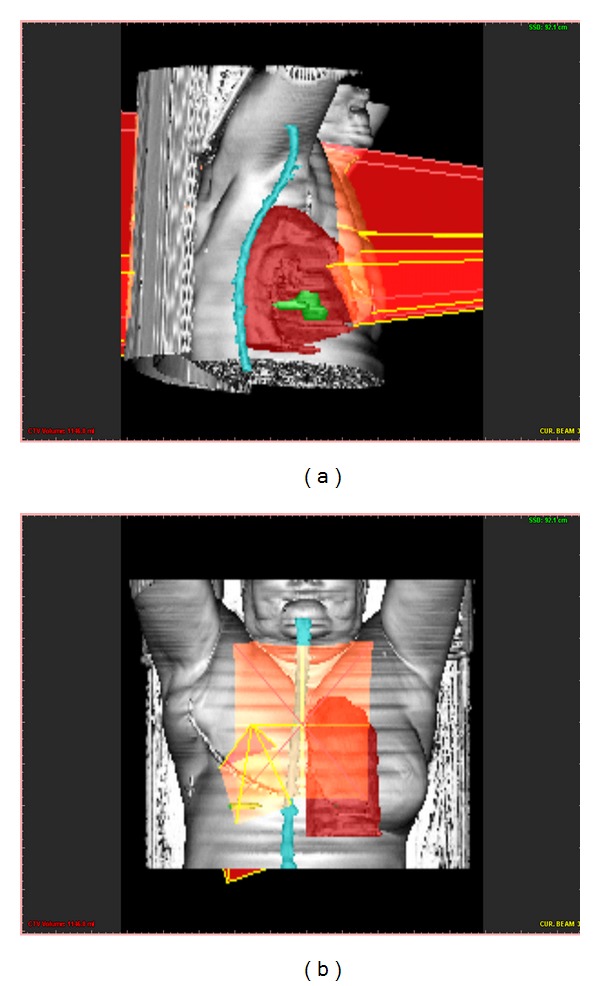
3D beam object reconstructed with patient's CT data. Green object: tumour.

Figure 15 illustrates examples where volume segmentation techniques and bean object reconstruction using Z-buffer methodology are applied.

To assess the effectiveness of the methods and its sensitivity, the following measures are considered: (1) area of a contour in cm^2^, (2) periphery of a contour in cm, (3) computational time in sec and (4) number of points for the contours drawing.  Table 1 illustrates the performance of the segmentation methods for various slices using the manual, the proposed method, and the proposed method after the point decimation process.

## 6. Volume Definition

Target volume and critical structure definition is a complex and time-consuming process in radiotherapy: although the complexity varies for different anatomical sites. In CT-Sim and plan evaluation, the physicists and radiation oncologists interact closely to subjectively identify the plan most appropriate for the individual patient. In order to reduce the investment of time and effort by the radiation oncology staff, several image analysis tools are integrated by CT-Sim systems. These systems thus allow the user to draw contours around the tumour as target [PTV] and around normal tissues and organs of interest on a slice-by-slice basis. This provides at the same time, a cross-reference to planar images.

**Figure 16:**
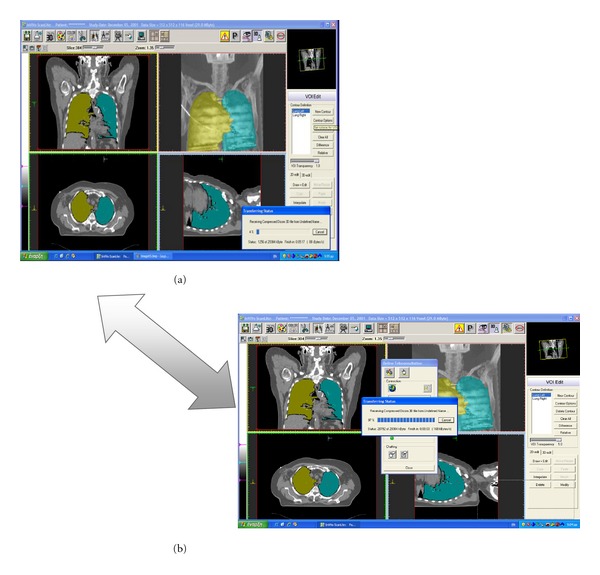
(a) Transformation of the reconstructed images under the telemedical platform. (b) Receiving process of the segmented medical images.

Organs with large differences in their radiation dose distributions can be segmented semiautomatically. In terms of user effort the only action required from the user is the selection of a starting point for the algorithm on the original axial slices. The complete 3D geometry of the organ will be traced automatically.  Figure 16 illustrates various applications of segmented anatomical structures under telemedical system.  Figure 16(a) illustrate transformation of the segmented medical images of the lungs.  Figure 16(b) illustrates the receiving process of the appropriate images.

Performance analysis of the system is taking place where the experiments are performed on a double processor (2xPentium III 450 MHz) PC with physical memory 512 Mb. For each view we performed time measurements for several rendering image sizes using different data resolution. The goal of this experiment is to examine the volume rendering interactivity as well as to prove the effectiveness of the system for large amount of data.

**Figure 17:**
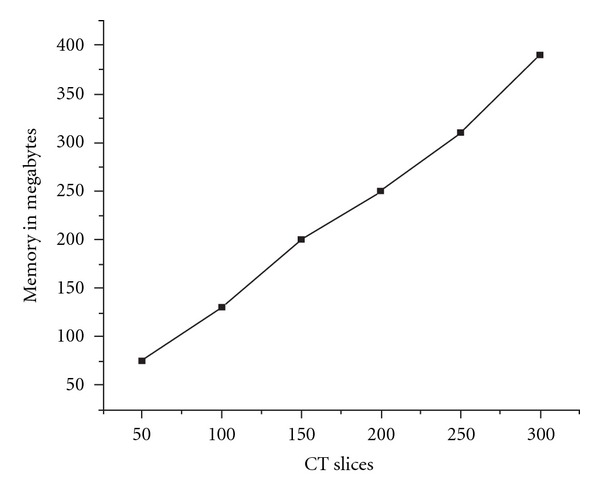
The system memory cost in the loaded CT slices.

A comparison for the system memory relative to different amount of data is presented in Figure 17. The results suggest a linear increase in required memory as it was expected. Important is that a PC with 512 MB physical memory can manipulate up to 300 slices with a rendering speed for transparent mode between 1.2 and 1.8 seconds and for the surface mode between 0.6 and 0.9 seconds.

**Figure 18:**
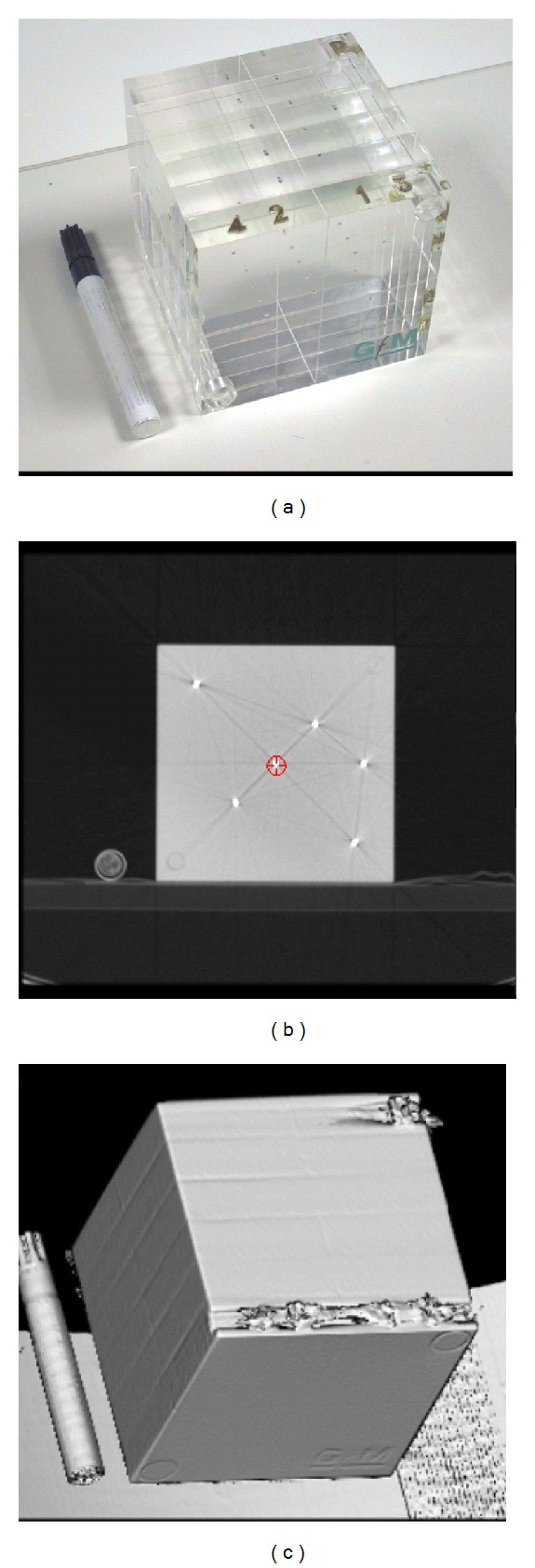
Phantom used for system verification. From (a)–(c) phantom photo, its CT scan, and its 3D surface reconstruction.

Accuracy of the system is very important for the system implementation especially for the investigation of (1) CT volume data orientation and (2) treatment parameters, including table translation, gantry, collimator, and table rotation, reference point placement, beam, and block size. Commonly slices thickness between 3 mm to 5 mm and total number of slices maximum to 120 slices are used to implement segmentation analysis or radiotherapy treatment planning dose. A cubic phantom of size 120 × 120 mm was initially used to exam the accuracy of the system. Inside the phantom spheres of diameter 2 mm are placed in constant distance. The phantom was scanned using 1 mm slice thickness. The total number of produced slices was 120, in square CT matrices of 512 × 512 pixels. The recorded system error concerning the table translation is at ±1 mm. The beam size and the projected light field size matched completely. No isocenter sphere effect occurred during component rotation. The interactive landmark registration routine might have the error of the slice thickness. The accuracy of the routines automatic field and block adaptation is related to the resolution of the BEV image (Figure 18). In most cases the BEV image has the size of 256 × 256 up to 400 × 400. The higher the image resolution the smaller the error of these routines.

## 7. Conclusions

CT-Sim is a technological advance that has already been tested in our clinical environment with demonstrable advantages particularly for complicated field arrangements. The new, innovative virtual simulator system is a very useful additional tool for the radiation oncologist and makes the requirement for a conventional X-ray simulator redundant. A significant advantage of the software is that it can run on portable computers such as laptops. This makes the CT-Sim concept extremely cost-effective. The system can be integrated immediately into the clinical environment via a direct interface to the CT scanner. The advantages of CT-Sim are not limited only to those mentioned above. Combination of different diagnostic (e.g., MR and PET) and radiotherapy (e.g., MV portal detectors) imaging modalities will make the CT-Sim an even more effective tool in radiotherapy. This technology could be combined with telemetrically techniques for the improvements of health services telemedicine applications are largely image-based. The main purpose of this work is to represent an efficient interactive, easy-to-use medical system that provides modules for online and offline collaboration of medical doctors together with software products. Moreover new improvements for the system starts to be implemented based on telemedical applications (this work is under development) where an online mode gives the opportunity to communicate over far distances with a given partner in real time. In this case both partners look at the same image material and through text messages (chat window) and transferred mouse actions to the remote PC, they can discuss interactively over a medical case. The system combines low price, low weight, mobility, and a wide range of nonradiating examinations. As a result the proposed platform reduces the disparity in the level of medical services, between the main centers and the less assisted areas, contributing to improvement of the quality of life.

## Figures and Tables

**Algorithm 1:**
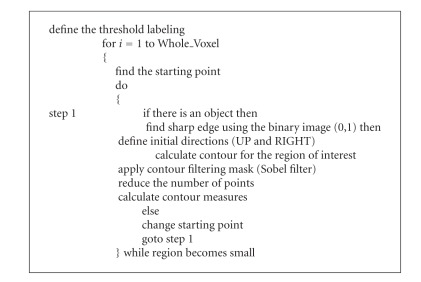


**Table 1:** Performance of the segmentation methods for various slices. M: manual; B: boundary tracking; D: boundary tracking after decimation.

	Area			Periphery			*≈*Time (sec)			Points		
Slices	M.	B.	D.	M.	B.	D.	M.	B.	D.	M.	B.	D.
22	37.6	37.5	32.2	41.6	55.7	50.1	40	5	2	600	530	424
42	72.4	70.2	68.1	52.4	61.5	62.3	60	5	2	850	712	569
62	59	56	52.8	30.8	40.8	38.9	40	5	2	540	418	334
